# Photo-catalytic Activities of Plant Hormones on Semiconductor Nanoparticles by Laser-Activated Electron Tunneling and Emitting

**DOI:** 10.1038/srep08893

**Published:** 2015-03-09

**Authors:** Xuemei Tang, Lulu Huang, Wenyang Zhang, Ruowei Jiang, Hongying Zhong

**Affiliations:** 1 Key Laboratory of Pesticides and Chemical Biology, Ministry of Education, College of Chemistry, Central China Normal University, Wuhan, Hubei 430079, P. R. China

## Abstract

Understanding of the dynamic process of laser-induced ultrafast electron tunneling is still very limited. It has been thought that the photo-catalytic reaction of adsorbents on the surface is either dependent on the number of resultant electron-hole pairs where excess energy is lost to the lattice through coupling with phonon modes, or dependent on irradiation photon wavelength. We used UV (355 nm) laser pulses to excite electrons from the valence band to the conduction band of titanium dioxide (TiO_2_), zinc oxide (ZnO) and bismuth cobalt zinc oxide (Bi_2_O_3_)_0.07_(CoO)_0.03_(ZnO)_0.9_ semiconductor nanoparticles with different photo catalytic properties. Photoelectrons are extracted, accelerated in a static electric field and eventually captured by charge deficient atoms of adsorbed organic molecules. A time-of-flight mass spectrometer was used to detect negative molecules and fragment ions generated by un-paired electron directed bond cleavages. We show that the probability of electron tunneling is determined by the strength of the static electric field and intrinsic electron mobility of semiconductors. Photo-catalytic dissociation or polymerization reactions of adsorbents are highly dependent on the kinetic energy of tunneling electrons as well as the strength of laser influx. By using this approach, photo-activities of phytohormones have been investigated.

Photo-catalytic reactions on the surfaces of semiconductors such as TiO_2_^1^,^2^,^3^ and ZnO^4^,^5^,^6^ have been extensively investigated since last century. The irradiation of laser pulses with energy more than semiconductor band gaps usually generates electron-hole pairs that would be available for either redox or oxidative chemistry^7^,^8^. However, charge carriers can only remain in the time scale of picoseconds or femtoseconds before they undergo fast thermalization^9^,^10^,^11^,^12^. Thus tremendous efforts have been focused on the development of time resolved transient spectroscopic techniques in order to understand fundamental mechanisms, which are important for applications in heterogeneous photocatalysis^13^,^14^, photosynthesis^15^,^16^,^17^ as well as environmental cleaning^18^,^19^,^20^.

It has been widely accepted that either numbers of electron-hole pairs^21^ or the photon energy (the wavelength)^22^ determines the dissociation rate of adsorbents on the surface of semiconductors. Because of rapid thermalization processes, photon generated electrons or holes have not been directly investigated. Instead, two photon photoemission^23^ and photoluminescence^24^ spectroscopic techniques have been used for such studies. With these approaches, Gundlach and coworkers^25^ initially detected a constant energy of emitted photons that is independent of the energy of exciting photons; Sporleder and coworkers^26^ found photo-desorption yield as well as photo-catalytic reaction rate are dependent on photon influx rather photon energy. Recently, Yang and coworkers^27^ demonstrated that photo-catalytic dissociation rate is actually highly dependent on photon energy. In any cases, direct experimental evidences how adsorbed organic molecules interact with photon generated electrons are important for judging the roles of photon energy or photon influx in photo-catalytic reactions. Measurement of emitting photons or yields and rates maybe limited for such purposes. We are thus aimed to directly monitor the dynamics of photon generated electrons and their interactions with adsorbed molecules. The basic experimental design includes a UV laser head (355 nm), an adjustable static electric field that can instantly extract and accelerate tunneling electrons as well as a time-of-flight mass analyzer that can detect ionized molecules and fragment ions. Gibberellic acid (Figure 1) with no conjugated unsaturated bonds for strong UV absorption was used as a model to study the photo-catalytic reactions on the surfaces of semiconductor nanoparticles by laser-activated electron tunneling (LAET) and emitting. Different semiconductor nanoparticles with different photo catalytic properties have been examined. Based on LAET mechanism, other plant hormones including salicylic acid, jasmonic acid, indoleacetic acid and abscisic acid have also been investigated.

## Methods

### Reagents and apparatus

LC-MS grade water, acetonitrile (ACN), ethanol and isopropanol were purchased from Fisher Scientific (NJ, USA). Nanoparticles of TiO_2_, ZnO, bismuth cobalt zinc oxide (Bi_2_O_3_)_0.07_(CoO)_0.03_(ZnO)_0.9_ (<100 nm BET or <50 nm XRD), gibberellic acid, salicylic acid, jasmonic acid, indoleacetic acid and abscisic acid were purchased from Sigma-Aldrich (MO, USA). Free fatty acids including C6:0, C8:0, C10:0, C12:0, C14:0, C16:0, C18:0, C20:0 and C22:0 were purchased from NU-CHEK PREP, Inc (MN, USA). All nanoparticles have been thermally treated at 350°C for 2 hours in a muffle furnace (Hubei, China) before use in order to remove trace organic contaminants.

### Detection of negatively charged ions

MALDI Synapt G2 HDMS system (Waters, USA) in negative mode was used to detect negatively charged molecular ions and fragment ions. It is equipped with an Nd: YAG high repetition laser head (355 nm). The laser influx (355 nm) has been set from 180 to 230 units. Laser pulse width is 3 ns and laser fire rate was set as 200 Hz. Pulse energy is 100 μJ/200 Hz. Potential differences between the sample plate and the aperture were adjustable and range from 20 volts to 100 volts. Voltages on the sample plate and aperture are 87 volts and 107 volts respectively for routine analysis. Changes in voltages can be conveniently performed on the MS Tune page of the instrumental control software. In negative mode, a mixture of free fatty acids including C6:0, C8:0, C10:0, C12:0, C14:0, C16:0, C18:0, C20:0 and C22:0 was deposited on surfaces of nanoparticles of semiconductors and used as lock masses for instrumental calibration. It should be noted that short and medium chain fatty acids (C6:0–C16:0) are evaporative. They may cause cross contamination with samples when they were spotted in the lock mass wells. In the case of gibberellic acid (GA), the mass spectra were calibrated with long chain fatty acid C18:0 in order to avoid ion suppression because GA generates fragment ions with masses very close to that of C6:0–C16:0. Nanoparticles of semiconductors were suspended in isopropanol solution and pipetted onto sample wells of the sample plate. Plant hormones such as Gibberellic acid or others were dissolved in a solution containing 50% acetonitrile and 50% ethanol to reach a concentration of 100 mg/mL. After nanoparticles on the sample plate were air dried, 1 μL of gibberellic acid solution or other solutions was deposited on the surface of nanoparticles. The sample plate was then put into the mass spectrometer and subjected to high vacuum.

## Results and Discussion

### Experimental design for extraction and acceleration of tunneling electrons

**Figure 1:**
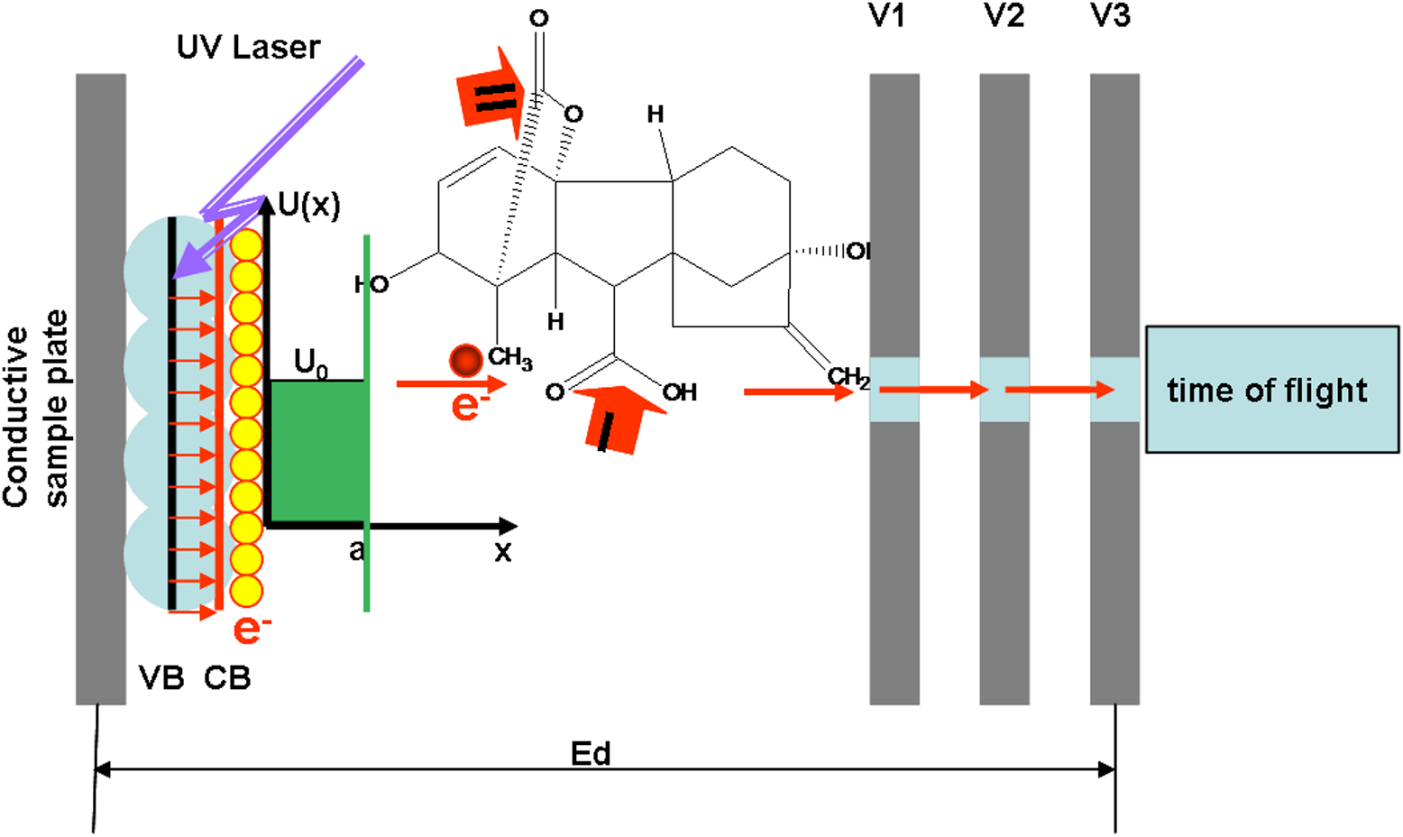
Instrumental set-up for detection of negative ions generated by laser activated electron tunneling and the structure of gibberellic acid. VB: valence band; CB: conduction band; UV: ultraviolet; Yellow balls: photo-generated electrons; Red balls: tunneling electrons; Blue balls: semiconductor nanoparticles; U(x): potential energy in x direction; U_0_: barrier energy; a: barrier width; x-axis: moving direction of electrons in the external electric field; V1-V3: voltages for extraction, hexapole and aperture respectively; Ed: strength of electric field. There are mainly two sites (I and II) for electron capture.

As shown in Figure 1, irradiation of ultraviolet (UV) laser pulses on the surface of semiconductors excites electrons in valence bands to conduction bands when the laser energy is more than the band gap. The potential barrier U_0_ prevents the escape of excited electrons away from the surface of nanoparticles. Tunneling probability for electrons to tunnel through the potential barrier can be described as equation (1) in which a represents the barrier width, m represents electron mass, E is the kinetic energy and 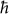 is Plank's constant^28^. It is shown that higher kinetic energy results in higher probability for electron tunneling and emitting. Equation (2)^29^ further indicates that the kinetic energy of electrons is correlated with electron mobility μ and the external electric field intensity E_d_. The presence of a static electric field not only instantly separates electron-hole pairs but also extract and accelerate electrons out of the surface of nanoparticles.
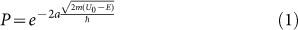

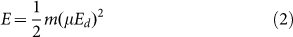
For routine mass spectrometric analysis, the potential difference between the sample plate and the aperture is about 20 volts at which the de Broglie wavelength of electrons does not match regular organic bonds (~0.14 nm). Kinetic energy of these electrons is about 20 eV that are not strong enough to cause intramolecular vibrational redistribution. However, they can be exothermically captured by charge-deficient atoms and generate negatively charged molecular ions as well as fragment ions resulting from un-paired electron directed bond cleavages. The kinetic energy of tunneling and emitting electrons are adjustable by changing the voltages on the sample plate or aperture. In this work, the potential differences between the sample plate and the aperture range from 20 volts to 100 volts while the irradiation wavelength is fixed at 355 nm. This experimental design provides a direct approach to investigate the fundamental mechanisms of photo-catalytic reactions on the surfaces of semiconductor nanoparticles. Compared with that of previously reported, this design is unique because it studies the photo-generated electrons instead of input photons or output photon emissions. The mass analyzer used in this work can only detect charged molecular ions or fragment ions. Then capture of negatively charged electrons and subsequent bond cleavages that switch neutral molecules to negatively charged ions can be monitored through the detection of corresponding ions.

### Photo-catalytic dissociation of gibberellic acid on the surface of nanoparticles of bismuth cobalt zinc oxide (Bi_2_O_3_)_0.07_(CoO)_0.03_(ZnO)_0.9_ and zinc oxide

**Figure 2:**
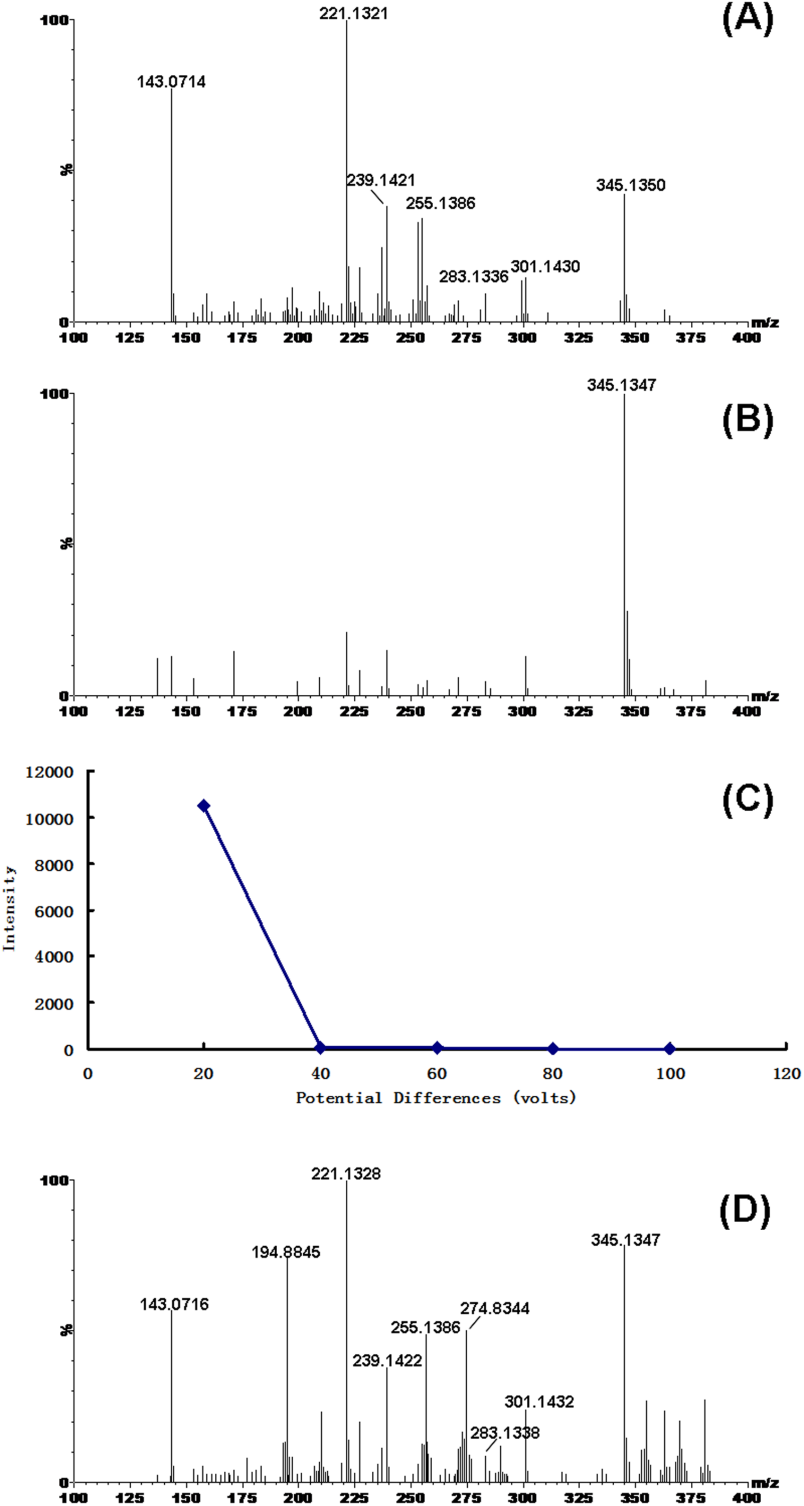
(A) Mass spectra of GA spotted on surfaces of bismuth cobalt zinc oxide and titanium dioxide (B) when the bias voltages between the sample plate and the aperture were 20 volts and the laser influx was set as 200 units.(C) Plot of absolute intensities of observed molecular ion at m/z 345 versus bias voltages when the laser influx was maintained as 200 units. (D) Mass spectrum of GA spotted on titanium dioxide when the laser influx was changed to 250 units but the bias voltage was maintained as 20 volts.

**Figure 3:**
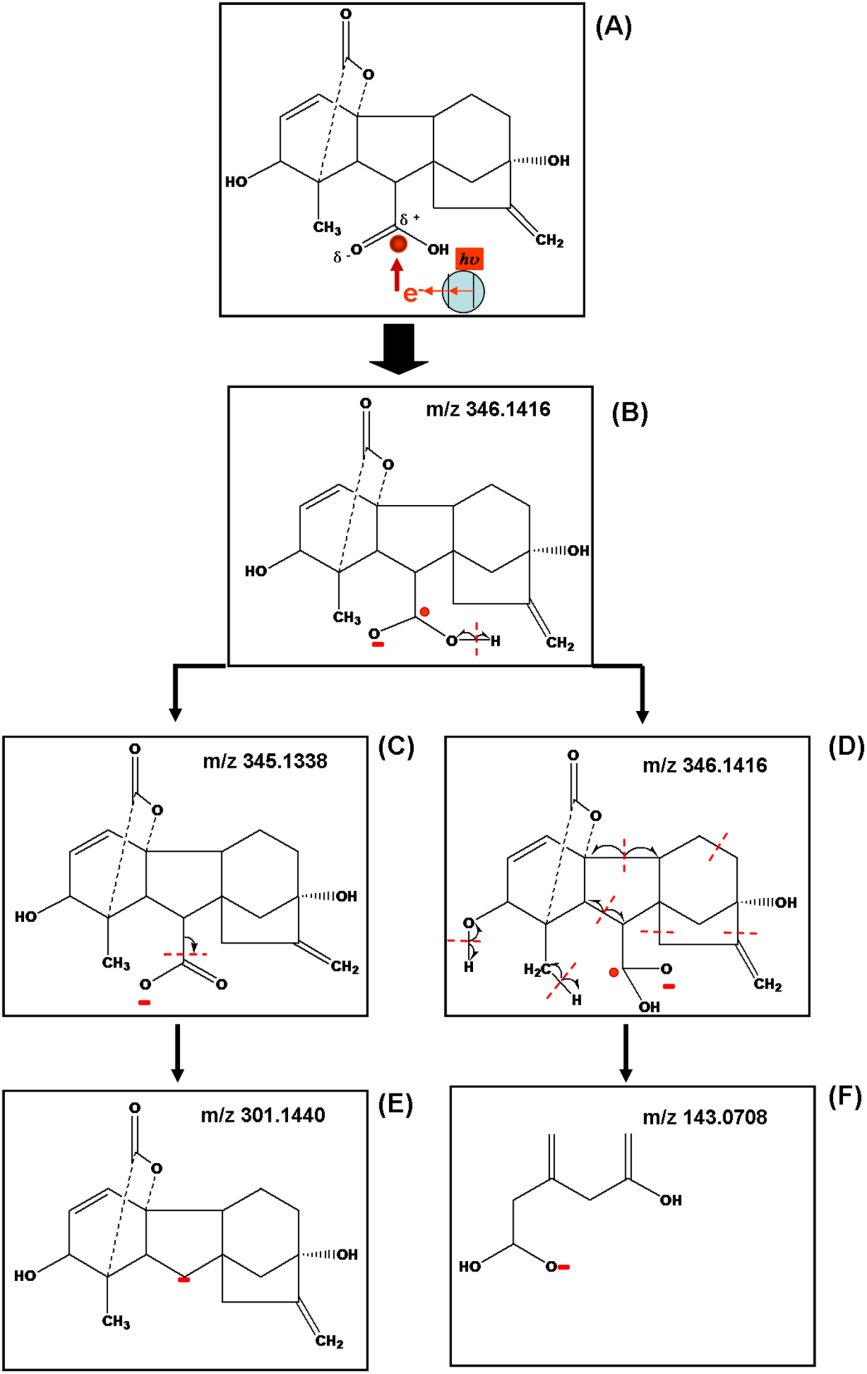
Capture of tunneling electrons by site I carbon atom of GA and unpaired electron directed chemical bond cleavages.

Gibberellic acid is one of the most important phytohormones (the structure is shown in Figure 1) that promotes growth and elongation of cells^30^. Its roles in plant physiology have been extensively investigated including stimulation of rapid stem and root growth^31^, induction of mitotic division in leaves of some plants^32^ and increases in seed germination rate^33^. This is the first time to demonstrate its photo-catalytic dissociation on the surfaces of semiconductor nanoparticles. Figure 2 (A) was mass spectra of GA spotted on surfaces of (Bi_2_O_3_)_0.07_(CoO)_0.03_(ZnO)_0.9_ nanoparticles. The spectrum was collected under a routine experimental condition where the potential difference between the sample plate and the aperture is 20 volts, the laser wavelength is 355 nm and the laser influx is set as 200 units. In Figure 2 (A), the most intense ions at m/z 143, 221, 239, 255 and 301 in addition to a molecular ion at m/z 345 (molecular ion M-H) attract the attention. Supplementary Figure 1 (A) shows that gibberellic acid does not have strong absorption within ultraviolet region (200 nm–400 nm). Supplementary Figure 2 shows that no signal of gibberellic acid or other fragment ions can be detected when laser pulses directly shot on gibberellic acid without the use of any semiconductor nanoparticles. The experimental results suggest that the ionization and fragmentation of gibberellic acid very likely result from the capture of laser-activated tunneling electrons and subsequent bond cleavages. A mechanism of multiphoton ionization was established in order to interpret observed ions. As shown in Figure 1, there are two carbonyl groups labeled as site I and site II respectively that can capture photo-generated electrons in gibberellic acid. Because of significant differences between electronegativities of carbon and oxygen, these carbonyl groups are moderately polar. As a result, the oxygen atoms have slight negative charges (δ^−^) and the carbon atoms have slight positive charges (δ^+^). In Figure 3 (A), the carbon atom of the carboxyl group in the neutral molecule (site I) captures one hot electron and produces an odd-electron hypervalent species (B). Resultant unpaired electron initiates α O-H bond cleavage and subsequent charge-directed C-C bond cleavage through non-ergodic processes. Two negatively charged species (C) and (E) are formed at m/z 345 and m/z 301 respectively. In addition to α O-H bond cleavage, the newly generated unpaired electron carried on species (D) also directs a series of other cleavages at α C-C bond along with H radical migrations through which a negatively charged species (F) at m/z 143 are produced.

**Figure 4:**
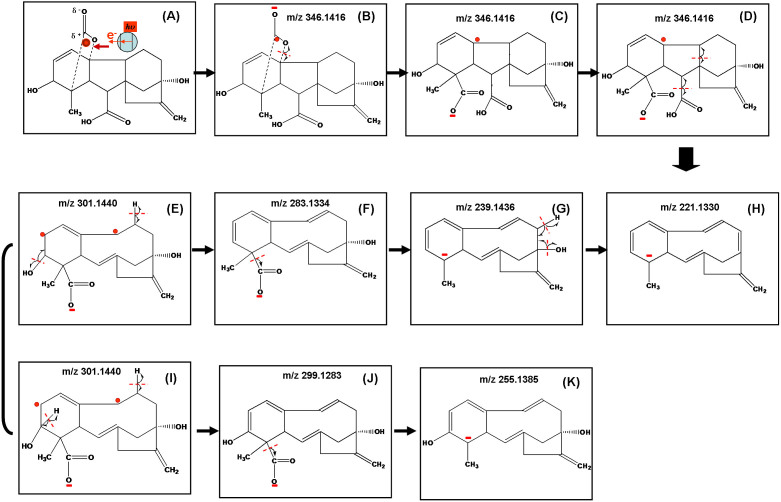
Capture of tunneling electrons by site II carbon atom of GA and unpaired electron directed chemical bond cleavages.

Capture of photo-generated electrons by site II carbon atom and subsequent bond cleavages were shown in Figure 4 (A). Through this way, another odd-electron hypervalent species (B) at m/z 346 can be formed. Species (C) was formed by unpaired electron-directed α C-O bond cleavage. Resultant new unpaired electron carried on species (C) further initiate sequential cleavages as shown in (D) by which several α C-C bond cleavages are involved. Starting from species (D), two kinds of products are eventually formed: (1) Species including (F) (m/z 283), (G) (m/z 239) and (H) (m/z 221) are generated through losses of H_2_O and CO_2_. (2) Species including (J) (m/z 299) and (K) (m/z 255) are generated through losses of H_2_ and CO_2_. In order to confirm the production of these ions, gibberellic acid spotted on the surface of (Bi_2_O_3_)_0.07_(CoO)_0.03_(ZnO)_0.9_ nanoparticles has been analyzed by the Q-TOF mass spectrometer in high resolution mode as shown in Figure 2 (A). Table 1 summarizes observed ions and their predicted masses. It was shown that all these predicted fragment ions can be detected with errors less than 6 ppm. These experimental results demonstrate that capture of photo-generated electrons causes specific unpaired electron-directed bond cleavages. The same mass spectrum has been obtained when GA was spotted on surfaces of ZnO nanoparticles as shown in Supplementary Figure 3.

By using the same technique, other plant hormones have also been investigated including indole-3-acetic acid (IAA), abscisic acid (ABA), jasmonic acid (JA) and salicylic acid (SA). Of these acids, SA shows distinguished photo activities on the surfaces of (Bi_2_O_3_)_0.07_(CoO)_0.03_(ZnO)_0.9_ nanoparticles which will be discussed in next section. Mass spectra of other three molecules were shown in Supplementary Figure 4. Molecular ions (M-1) and other fragment ions similar to that of GA have been observed.

In summary, capture of additional electrons is the key starting-up point for photo-catalytic reactions through which unpaired electrons can initiate a series of bond cleavages. Although it has been well recognized that photo-generated holes usually produces hydroxyl radical ions that are the most oxidative species, this work has discovered that photo-generated hot electrons actually take the major roles in photo-catalytic dissociation reactions when photocatalytic reactions occur in a high vacuum environment.

### Photo-catalytic dissociation of gibberellic acid on the surface of nanoparticles of titanium dioxide TiO_2_

In order to further explore activities of photo-generated electrons, gibberellic acid has been spotted on TiO_2_ nanoparticles whose band gap is similar as that of zinc oxide but electron mobility is about 20 times lower. However, all other experimental conditions including laser wavelength and strength of electric field voltages were maintained the same as that described for zinc oxide. It was found in this experiment that less fragmentation can be observed when GA was spotted on surfaces of TiO_2_ nanoparticles. As shown in Figure 2 (B), the molecular ion (M-1) at m/z 345 is the dominant peak and other fragment ions are relatively very much low abundance. Compared with ZnO, TiO_2_ has much lower electron mobility and thus much lower tunneling probability^29^. It has been discovered in our previous work that decreased kinetic energy resulting from decreased electron mobility not only causes decreased tunneling probability but also decreased fragmentations. The result shown in Figure 2 (B) confirms this discovery once again. However, under this situation, fragmentation can still be achieved by increasing the bias voltage between the sample plate and the aperture because photo-generated electrons are accelerated^29^. But it should be mentioned that different fragmentation mechanisms maybe involved and different fragment ions maybe obtained. Supplementary Figure 5 demonstrates the roles of external electric field in kinetic energy, electron impact and intramolecular vibrational energy redistribution-associated molecular fragmentation. Fragment ions observed in this figure are different from that of Figure 2 (A). In Supplementary Figure 5 (A–E), potential differences between the sample plate and the aperture were changed from 20 volts to 100 volts respectively. At 40 eV high energy, the de Broglie wavelength of electrons is more than regular bond length of organic molecules. So these electrons may be strong enough to cause intra-molecular vibrational redistribution of energy. Additionally, because their energies are higher than typical ionization potential (7~15 eV), the impact of these hot electrons on organic molecules may be able to kick out lone-pair electrons and initiate extensively subsequent fragmentation. It is shown that the molecular ion at m/z 345 dramatically decreases but several other fragment ions can be observed when the potential difference increases to 40 volts. Further increases in potential differences result in even decreased intensity of the molecular ion at m/z 345 as summarized in Figure 2 (C). This experimental result raises attention to different consequences of two different photo-generated electrons: (1) When bismuth cobalt zinc oxide nanoparticles are used, there is high tunneling probability because of high electron mobility. Kinetic energy obtained from external electric field is only about 20 eV. Therefore ionization of these molecules is caused by exothermic capture of tunneling electrons. (2) When titanium dioxide nanoparticles are used, there is low tunneling probability because of low electron mobility. While increased external electric field increases tunneling probability, these tunneling electrons are also accelerated in the external electric field. Kinetic energy obtained from external electric field is 40 eV or even higher that is high enough to kick out electrons with low ionization potentials such as lone-pair electrons. So ionization of these molecules may be caused by the loss of electrons. Additionally, at 40 eV or higher energies, de Broglie wavelength of electrons is more than organic bond length. These electrons are then able to cause intramolecular vibrational redistribution that may causes extensive non-specific bond cleavages or rearrangements. Taken together, although all these photo-catalytic reactions are caused by unpaired electrons in both two cases, exothermic capture of electrons is mild while electron impact is energetic and produces much more non-specific fragment ions. Elsewhere, stabilities of parent ions and product ions also play important roles in fragmentation pathways. This is in accordance with what have been observed. In addition to electron kinetic energies, we have further demonstrated in this work that fragmentation can also be achieved by increasing the strength of laser influx as shown in Figure 2 (D) where the laser influx was increased from routine 200 units to 250 units.

All the experimental results demonstrate two important issues: (1) Increased strength of laser influx (or photon influx) actually increases the number of photo-generated electron-hole pairs because more electrons in valence bonds are excited to conduction bonds. Subsequently the number of tunneling electrons as well as electron captures and electron-directed fragmentations increase. Therefore, stronger intensities of fragment ions can be detected as shown in Figure 2 (C). (2)Laser activated electron tunneling and electron capture is a comprehensive photo-catalytic process that is associated with several factors. First, the laser wavelength determines if electrons in valence bond can be excited to conduction bond. The influx photon energy should be more than that of semiconductor band gaps. Secondly, the kinetic energy of photo-excited electrons determines the probability if tunneling away from the potential barrier can occur. But the kinetic energy is determined by intrinsic electron mobility and external electric field. Thirdly, tunneling electrons can either be exothermally captured by charge deficient atoms of adsorbed organic molecules or accelerated in the external static electric field and kick out electrons with lower ionization potentials such as lone pair electrons of adsorbed molecules. Different electron fates determine different fragmentation pathways. Finally, the strength of influx laser determines the number of excited electrons and thus the intensities of product ions. Actually, there are also many other factors that may determine photo-catalytic reaction rates such as charge carriers, recombination of photogenerated electron-hole pairs, crystallinity and particle sizes of photocatalysts. This work is only focused the studies of interactions of photo-generated electrons and adsorbed organic molecules.

### Photo-catalytic polymerization of gibberellic acid on the surface of bismuth cobalt zinc oxide (Bi_2_O_3_)_0.07_(CoO)_0.03_(ZnO)_0.9_ nanoparticles

**Figure 5:**
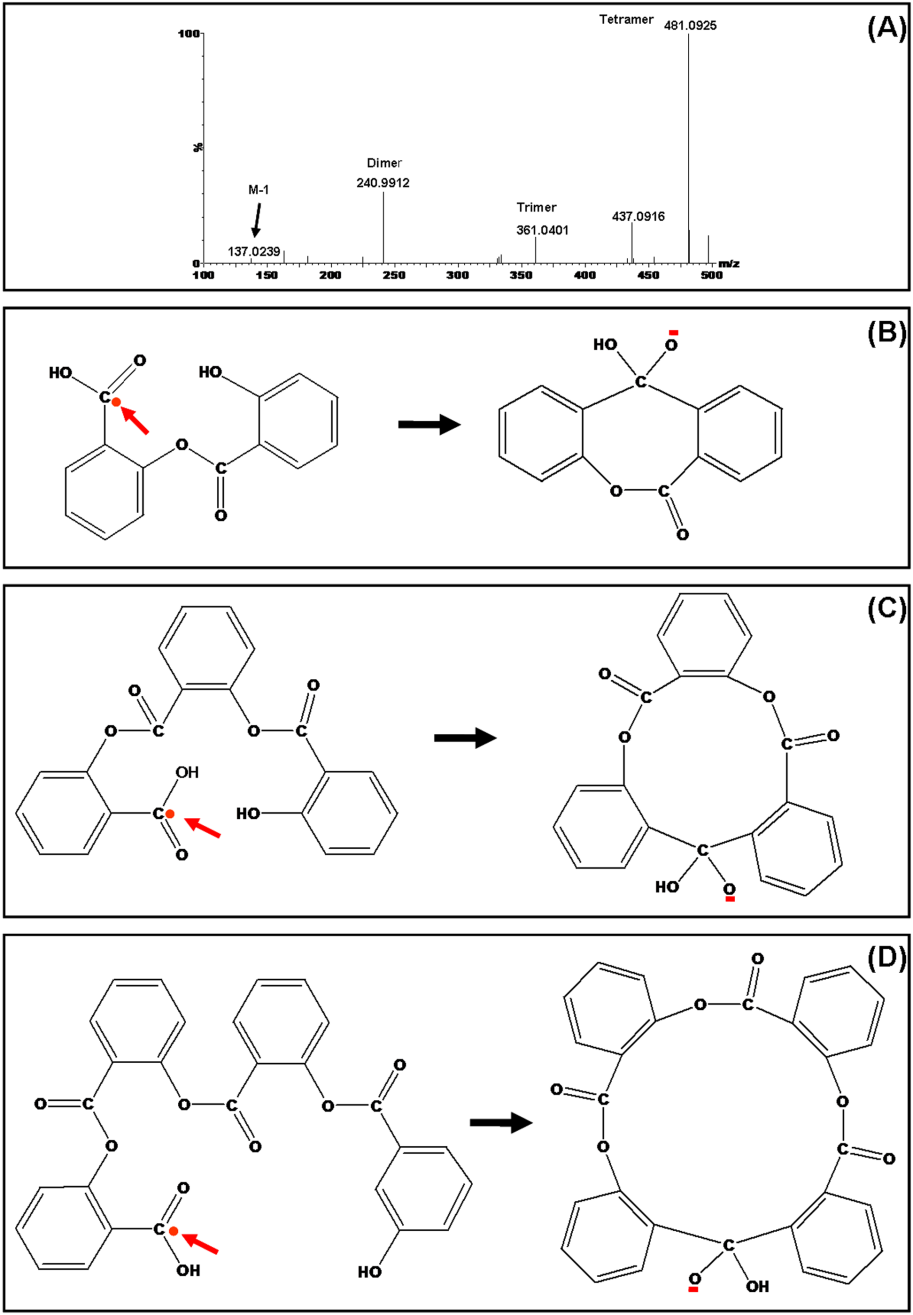
Mass spectrum of photo-catalytic polymerization of SA on surfaces of bismuth cobalt zinc oxide nanoparticles (A) and formation of negatively charged dimeric (B), trimeric (C) and tetrameric (D) ions.

While photo-catalytic dissociation reactions have been extensively studied, less attention has been paid to photo-catalytic polymerization in the past. We provide here experimental evidences that can demonstrate the formation of dimeric, trimeric and tetrameric ions of salicylic acid (SA) on the solid surfaces of (Bi_2_O_3_)_0.07_(CoO)_0.03_(ZnO)_0.9_ semiconductor instead of gas phase. As shown in Figure 5 (A), in addition to the molecular ion at m/z 137 as well as other low abundance peaks at m/z 241 and 361, an ion at m/z 481 dominates the mass spectrum. These peaks were considered as the results of photo-catalytic polymerization and the mechanisms were interpreted as Figure 5 (B), (C) and (D) for the formation of dimeric, trimeric and tetrameric ions of SA respectively. Presence of benzene ring in SA makes it have strong absorption in UV region shown in Supplementary Figure 1 (B). Formation of ester bonds between OH and COOH results in the linkage of two, three or four neutral salicylic acids. As illustrated in Figure 5 (B–D), laser activated hot electrons can be captured by charge deficient carbon atoms of carboxylic groups of the polymers of salicylic acid. Subsequent tandem processes include unpaired electron-directed intermolecular substitutions and the formation of negatively charged poly-membered rings with the loss of OH radical ions. The ion at m/z 481 is the largest macrocyclic ion we have detected so far. It shows the strongest stability among all these macrocyclic ions. Ion mobility experiments demonstrate that these ions have different collision cross sections as shown in Supplementary Figure 6. In order to investigate if these ions were formed in the gas phase or on the solid surfaces of nanoparticles, different semiconductors have been studied. It was found that SA cannot undergo polymerization when TiO_2_ or ZnO material was used as shown in Supplementary Figure 7. This experimental result indicates that the photo-catalytic polymerization reaction indeed occurs on the solid surfaces. Dopping of Bi_2_O_3_ in ZnO changes the photo-catalytic properties.

Additionally, it should be pointed out that SA is very evaporative under vacuum condition. We have found that SA may cause extensive cross contamination when it was spotted on the same sample plate even it is very far away from analyzes as shown in Supplementary Figure 5. With high concentration, it may completely suppress the signal of analyzes. Therefore, all experiments of SA should be performed separately.

## Conclusion

Our laser-activated electron tunneling investigation with time-of-flight mass spectrometer provides experimental evidences that photo-catalytic dissociation or polymerization on the surface of semiconductors is highly dependent on kinetic energies of tunneling electrons in addition to the influx of laser pulses. When the wavelength of laser irradiation is fixed at 355 nm, excitation probability of electrons from valence bands to conduction bands is similar if there are similar band gaps for different materials such as TiO_2_ and ZnO. Higher tunneling probability results from higher kinetic energy of tunneling electrons that is determined by intrinsic electron mobility and external electric field. Adsorbed organic molecules can be ionized through exothermic capture of tunneling electrons and resultant unpaired electrons initiate subsequent bond cleavages or linkages. Although electrons with low kinetic energy can be accelerated in the external electric field, extra energies more than ionization potential cause losses of lone-pair electrons as well as intramolecular vibrational redistribution which eventually results in many fragment ions through different mechanisms and pathways. Compared with widely accepted model of photo-catalytic reactions on the surface of semiconductors, this work reveals the roles of photo-generated hot electrons in addition to photon influx or wavelength. It provides an experimental approach for direct investigations on interactions of photo-generated electrons with adsorbed molecules. Although hydroxyl radical ion ·OH has been assumed as the most oxidative species in photo-catalytic reactions, we have demonstrated that exothermal capture of photo-generated electrons actually plays important roles in photo-catalytic reactions under high vacuum condition.

Gibberellic acid that does not have conjugated unsaturated bonds for strong UV absorption has been used as the model to explore the mechanisms of photo-catalytic reactions on the surface of semiconductors. It is intriguing attractive to see that gibberellic acid, salicylic acid as well as other three plant hormones have distinguished photo-catalytic properties on surfaces of semiconductor nanoparticles with UV laser irradiation, which maybe important for understanding of photo sensitive actions.

## Author Contributions

H.Z. developed the concept, designed the experiments, analyzed the data, and wrote the manuscript. X.T. and L.H. performed experiments and repeated with each other. W.Z. drew art works and has been involved in part of experiments. R.J has been involved in part of experiments.

## Supplementary Material

Supplementary Information Supporting Information

## Figures and Tables

**Table 1:** Ionization and fragmentation of gibberellic acid based on LAET

Molecular formula	Expt (Da)	Obsd (Da)	Error (Da)
C_19_H_21_O_6_	345.1338	345.1350	0.0012
C_18_H_21_O_4_	301.1440	301.1430	0.0010
C_18_H_19_O_4_	299.1283	299.1272	0.0011
C_18_H_19_O3	283.1334	283.1336	0.0002
C_17_H_19_O_2_	255.1385	255.1386	0.0001
C_17_H_19_O	239.1436	239.1421	0.0015
C_17_H_17_	221.1330	221.1321	0.0009
C_7_H_11_O_3_	143.0708	143.0714	0.0006
