# Supplementary material for: Photo-catalytic Activities of Plant Hormones on Semiconductor Nanoparticles by Laser-Activated Electron Tunneling and Emitting
Source: Sci Rep. 2015 Mar 9;5:8893. doi: 10.1038/srep08893 (PMC4352873; doi:10.1038/srep08893)
Supplement: Supplementary Information — Supporting Information [file srep08893-s1.doc]

**Photo-catalytic Activities of Plant Hormones on Semiconductor Nanoparticles by Laser-Activated Electron Tunneling and Emitting**

**Xuemei Tang^+^, Lulu Huang^+^, Wenyang Zhang, Ruowei Jiang, Hongying Zhong^*^**

**Key Laboratory of Pesticides and Chemical Biology, Ministry of Education, College of Chemistry, Central China Normal University, Wuhan, Hubei 430079, P. R. China**

*** To whom correspondence should be addressed. Email:** [**hyzhong@mail.ccnu.edu.cn**](mailto:hyzhong@mail.ccnu.edu.cn)**, Tel: 86-27-67862616**

**^+^ These authors contribute equally to this work.**
